# Pandora's Box

**DOI:** 10.1192/bji.2021.9

**Published:** 2021-05

## Abstract

We humans thrive on social contact, and conversation is our main means of interaction. Many of us can recall long conversations and having difficulty putting an end to these, but also other times when we may feel aggrieved that the conversation has come to a premature end. We think we can read others’ facial expressions and body language, but perhaps we're not so good at this as we think.

## Keep talking…

We humans thrive on social contact, and conversation is our main means of interaction. Many of us can recall long conversations and having difficulty putting an end to these, but also other times when we may feel aggrieved that the conversation has come to a premature end. We think we can read others’ facial expressions and body language, but perhaps we're not so good at this as we think.

Researchers at Harvard University examined this very subject in two studies involving 932 conversations. They asked the index conversants to report when they wanted to end a conversation, and to also estimate when they believed their fellow conversant would want to end the conversation. The conversation partner was a person intimately known to the index conversant in Study 1 and a stranger in Study 2. Interestingly, the results showed that conversations almost never ended at the time that both conversants wanted them to end and rarely ended when one conversant wanted them to end. The desired duration of the conversation was half of the actual duration. The conversants had very little clue when the other person wanted to end the conversation, irrespective of whether (s)he was an intimate person or a stranger.

Mastroianni AM, Gilbert DT, Cooney G, Wilson TD. Do conversations end when people want them to? PNAS (2021) 118(10), e20211809118.10.1073/pnas.2011809118PMC795823133649209

## Is marital bliss down to our genes?

Why is married life satisfying and happy for some couples, but for others the relationship declines in the first few years of marriage? Could it be down to our genes? Research over recent years indicates the importance of genetic variation on the CD38 gene; the single-nucleotide polymorphism rs3796863 is associated with cognition and behaviour in rodents and with positive outcomes in human romantic relationships that relate to pair bonding. The same gene is also known to code for the transmembrane glycoprotein CD38, which has multiple functions throughout our body, one of which is the modulation of oxytocin release.

On the above basis, and replicating and extending previous evidence, researchers obtained longitudinal data from 71 newlywed couples who were genotyped, and examined whether rs3796863 bore any association with processes involved in maintaining a relationship and with relationship satisfaction in the early years of marriage. They found that individuals with the CC genotype of rs3796863 reported higher levels of gratitude, trust and marital satisfaction compared with those with the AC and AA genotypes. Furthermore, the benefits for CC individuals lasted over the whole of the first 3 years of marriage.

It may be worth undergoing genetic testing prior to marriage; this may save many people a lot of heartache, acrimony and costly divorce proceedings!

Makharova A, McNulty JK, Eckel LA, Nikonova L, Bartz JA, Hammock EAD. CD38 is associated with bonding-relevant cognitions and relationship satisfaction over the first 3 years of marriage. Sci Rep 2021; 11: 2965.3353648910.1038/s41598-021-82307-zPMC7859203

## The Amazons of the early Americas

The 8 March 2021 marked yet another anniversary of International Women's Day and the striving for gender parity. Women have been led to believe that from the very beginnings of human existence, men were the hunters and women the gatherers. These gender roles continued and developed over the following thousands of years to the present. Men traditionally were the providers, the protectors of the family, and women's role was to keep a home, reproduce and care for the offspring. Technology liberated women, and finally we are getting close to some form of gender parity.

Recent archaeological evidence turns upside-down these earlier assumptions on gender roles. Excavations at Wilamaya Patjxa, a highland in the Andes in South America, revealed a 9000-year-old human burial site containing a hunting toolkit of stone projectile points and animal processing tools. Interestingly, the human body of the hunter found alongside the tools was identified not as a male but as a young adult female, who according to osteological proteomic and isotopic analysis subsisted on terrestrial plants and animals. Other burial sites of the Late Pleistocene and Early Holocene across the Americas included 10 other females and a similar number of early males in hunter burial sites. These findings are consistent with labour practices at the time, including big game hunting, being shared by both men and women.

Over the following thousands of years, women gradually assumed less risky and less hard physical work, which, although welcome, was associated with the gradual loss of gender parity and subordination of women.

Haas R, Watson J, Buonasera T, Southon J, Chen JC, Noe S,  Female hunters of the early Americas. Sci. Adv. 2020; 6(45): eabd0310.10.1126/sciadv.abd0310PMC767369433148651

## A walk in the park every day keeps amphetamines at bay

We have finally come to appreciate the major role of greenery in our physical and psychological well-being. What we have failed to appreciate so far is the importance of green spaces on our children's developing brains.

Researchers at Aarhus University in Denmark examined the possible effects of green spaces on children in relation to attention-deficit hyperactivity disorder (ADHD), a common psychiatric diagnosis among children that often requires long-term amphetamine-type medication. In a nationwide cohort study, they investigated associations between residential green spaces in early childhood and a diagnosis of ADHD. A cohort of over 800 000, which included individuals born in Denmark between 1992 and 2007, was followed for diagnosis of ADHD from age 5 during the period 1997 to 2016. The researchers measured the amount of vegetation greenness surrounding each residential address using the normalised difference vegetation index (NDVI). The average NDVI surrounding each individual's residential address from birth to their 5th birthday was the measure of individual exposure to green space. Using a multilevel modelling analysis, they estimated incidence rate ratios with 95% confidence intervals for ADHD according to green exposure level. They adjusted for calendar time, age and sex, as well as parental and neighbourhood socioeconomic status and urbanicity.

Their results indicated that individuals who lived in areas with sparse green vegetation, i.e. the lowest decile of NDVI, had an increased risk of developing ADHD compared with individuals who lived in areas within the highest decile of NDVI.

Thygesen M, Engemann K, Holst GJ,  The association between residential green space in childhood and development of attention deficit hyperactivity disorder: a population-based cohort study. Environ Health Perspect 2020; 128: 127011. doi: 10.1289/EHP6729.33351671PMC7755168

## The placebo effect – not as we know it

There is no dispute about the effectiveness of placebo, and this is taken into consideration in both research and clinical practice. However, despite the fairly large number of studies, mainly on the subject of pain, it has not been possible to pinpoint the underlying neurobiological mechanisms involved. A recent publication in *Nature Communications* went some way towards identifying the brain systems involved in placebo analgesia.

The authors carried out a meta-analysis of experimental functional magnetic resonance studies of evoked pain, under stimulus intensity-matched, placebo and control conditions, which included 603 healthy participants from 20 studies. Mapping the effects of placebo treatment on pain-related brain activity, they identified neural correlates of individual differences in behavioural placebo analgesia. Their results corroborated previous findings of increases in activity in the frontal-parietal regions and reductions in the insula, as well as effects in new areas not previously reported; these included reductions in activity in the habenula, specific parts of the thalamus and the cerebellum.

The conclusion is that placebo treatment affects pain-evoked activity in multiple brain areas, which are involved not only in nociception but also in other affective and decision-making processes related to pain perception. Should we clinicians be making more use of the placebo effect? One could ponder whether in the current litigation-prone climate, we have perhaps become less effective. Presenting our patients with a treatment plan, be it surgical, pharmacological or psychological, are we too restrained in conveying the chosen therapy in a more positive light rather than a neutral, even cold, self-protective, don't blame me if it doesn't work?

Zunhammer M, Spisak T, Wager TD, Bingel U, Placebo Imaging Consortium. Meta-analysis of neural systems underlying placebo analgesia from individual participant fMRI data. Nat Commun 2021; 12: 1391.3365410510.1038/s41467-021-21179-3PMC7925520

## Being fanatic is not clever

The recent events surrounding the US presidential elections and the storming of the Capitol have highlighted the dangerousness of extreme ideologies and fanaticism. It is part of our humanity to hold a variety of ideologies, and some of us can take this to extremes. There are differences among us in how we perceive information and how we process this to inform and develop our individual views of the world. What makes some of us more prone to extremist beliefs, which are resistant to change despite evidence? Pandora has discussed in previous issues the findings of studies on this subject.

A recent study from Cambridge University aimed to uncover the specific psychological signatures of political, nationalistic, religious and dogmatic beliefs. The researchers used a much larger than usual number of cognitive tasks (n = 37) and personality surveys (n = 22), together with data-driven analyses, which included drift diffusion and Bayesian modelling. They found that cognitive and personality assessments were 4–15 times more predictive than demographic predictors of individual differences in ideological preferences. They also found that individual ideological attitudes mirrored the participant's cognitive decision-making approaches. They claim to have uncovered specific psychological signatures. Conservatism and nationalism were associated with greater caution in perceptual decision-making tasks and strategic information processing. Dogmatism was associated with slower evidence accumulation and impulsive tendencies. Religiosity was associated with heightened agreeableness and risk perception. They also found that extreme group attitudes, which included endorsement of violence towards outgroups, were linked to poor working memory and tendencies towards impulsivity and sensation-seeking. These individuals also exhibited slower perceptual strategies, showing similarities to the psychological profiles of conservatism and dogmatism.

Overall, these findings suggest that our ideological worldviews may reflect our individual psychological processing and that lower-level perceptual and cognitive functions may allow the development of toxic ideologies. By contrast, those with cognitive traits that make them more receptive to evidence are more resilient to extremist rhetoric. The authors claim that the identified relationship between ideology and cognition offers an invaluable foundation for research into the psychological roots of intergroup attitudes, xenophobia and ideological extremism.

Zmigrod L, Eisenberg IW, Bissett PG, Robbins TW, Poldrack RA. The cognitive and perceptual correlates of ideological attitudes: a data-driven approach. Philos Trans R Soc B 2021; 376(1822): 20200424.10.1098/rstb.2020.0424PMC793510933611995

